# Executive Function Deficits in Seriously Ill Children—Emerging Challenges and Possibilities for Clinical Care

**DOI:** 10.3389/fped.2018.00092

**Published:** 2018-04-18

**Authors:** Annet Bluschke, Maja von der Hagen, Barbara Novotna, Veit Roessner, Christian Beste

**Affiliations:** ^1^Cognitive Neurophysiology, Department of Child and Adolescent Psychiatry, Faculty of Medicine, Technische Universität Dresden, Dresden, Germany; ^2^Abteilung Neuropädiatrie, Medizinische Fakultät Carl Gustav Carus, Technische Universität Dresden, Dresden, Germany; ^3^Experimental Neurobiology, National Institute of Mental Health, Prague, Czechia; ^4^Institute of Psychology, Technische Universität Dresden, Dresden, Germany

**Keywords:** cognitive dysfunctions, executive function, therapy, cognitive training, chronic disease

## Abstract

The past years have seen an incredible increase in the quality and success rates of treatments in pediatric medicine. One of the resulting major challenges refers to the management of primary or secondary residual executive function deficits in affected children. These deficits lead to problems in the ability to acquire, understand, and apply abstract and complex knowledge and to plan, direct, and control actions. Executive functions deficits are important to consider because they are highly predictive of functioning in social and academic aspects of daily life. We argue that current clinical practice does not sufficiently account for the complex cognitive processes in this population. This is because widely applied pharmacological interventions only rarely account for the complexity of the underlying neuronal mechanisms and do not fit well into possibly powerful “individualized medicine” approaches. Novel treatment approaches targeting deficits in executive functions in seriously ill children could focus on neuronal oscillations, as these have some specific relations to different aspects of executive function. Importantly, such treatment approaches can be individually tailored to the individuals’ deficits and can be transferred into home-treatment or e-health solutions. These approaches are easy-to-use, can be easily integrated into daily life, and are becoming increasingly cost-effective.

## Introduction

**Figure 1:**
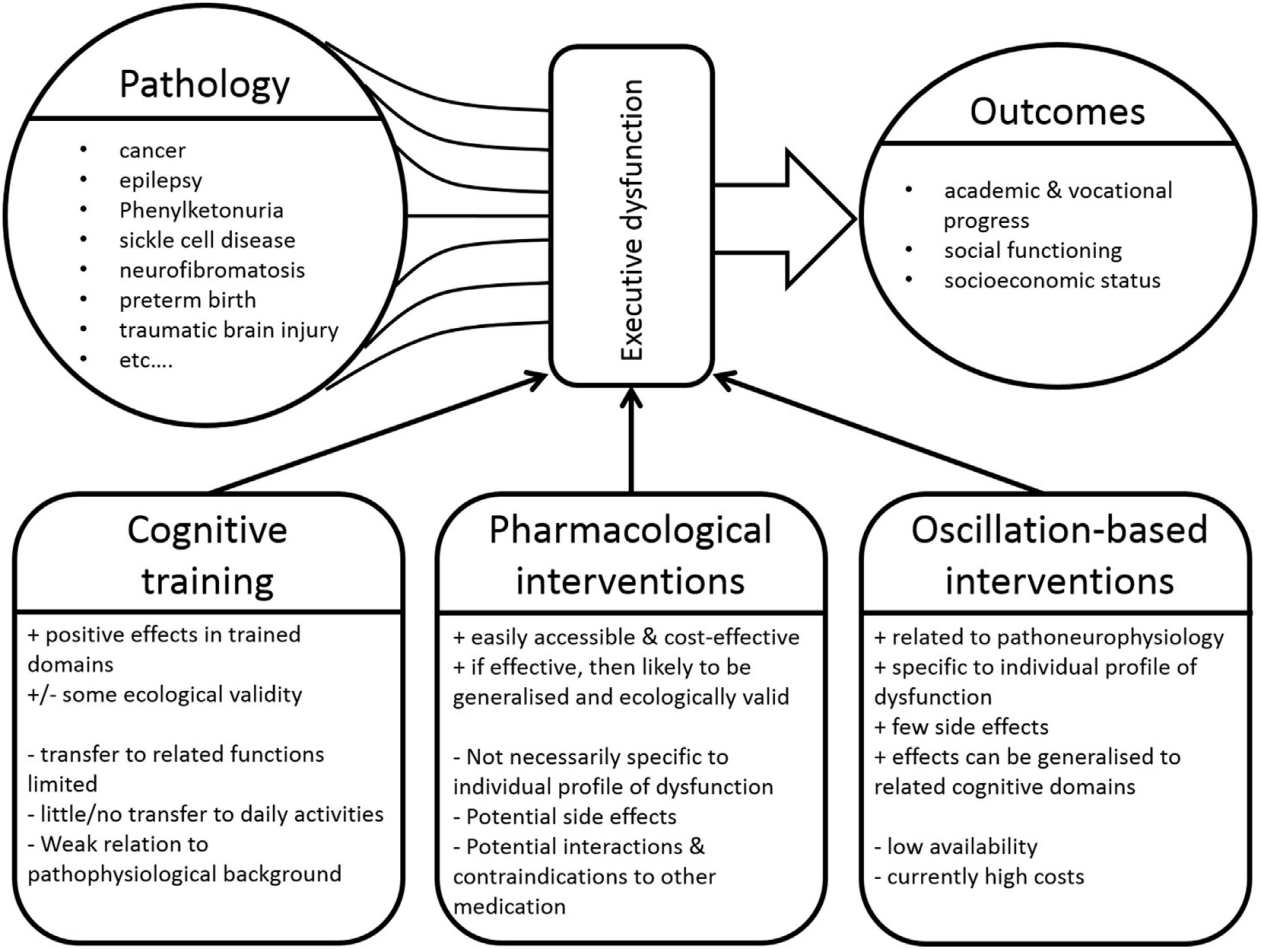
A large number of diseases can result in executive function deficits, which in turn significantly influence a variety of outcome variables. Various approaches can be applied to address such deficits. “+” denotes advantages of these intervention approaches, whereas “−” denotes disadvantages.

In recent years, advances in pediatric medicine have led to significantly higher survival rates of children with serious acute or chronic life-limiting conditions (1–4). In particular, the option of an individual adjustment of treatment to the particular characteristics of a particular disease in a particular patient (5) has opened new avenues for treating clinicians. These treatment advances have, however, resulted in an increasing group of children and (young) adult patients with residua or sequelae of (often rare) childhood diseases. This introduces new problem areas, since increased survival is resulting in new morbidities that may significantly affect psychosocial outcomes (1, 6–8). Concerning this issue, questions relating to general intellectual abilities in these children and the development of various cognitive functions are moving more and more into the focus (9). To name just a few, problems in cognitive functioning that are frequently associated with various (neuro)psychiatric/developmental disorder have been reported to occur as direct primary effects or as secondary results of a number of devastating diseases. These include, but are not limited to, cancer (10, 11), congenital, and chronic developmental disorders affecting the nervous system like epilepsy (12), sickle cell disease (13), spina bifida (14), phenylketonuria (15), and neurofibromatosis type 1 (16) as well as incidents significantly interfering with development like preterm birth (17) or traumatic brain injury (18, 19) (see Figure 1). We suggest that, just like it is the case for the underlying condition, such problems should be addressed through therapeutic strategies which strongly take the individual characteristics of deficits of the affected patients into account (see Table 1 and Figure 1).

**Table 1:** Comparison of cognitive (executive function) training, pharmacological interventions and oscillation-based interventions on different dimensions currently defining individualized/personalized medicine [adapted from Ref. (5)].

Possible interventions for executive dysfunctions				
Dimension	Does the treatment…	Cognitive training	Pharmacological interventions	Oscillation-based interventions
Disease	…consider the specific cognitive deficits?	++	+	++
Patient’s environment	…consider environmental aspects of cognitive deficits?	++	–	+
Genes	…consider how genetic factors influence the cognitive deficits?	–	–	–
Medication/Neurobiology	…target the specific neurobiological basis of the cognitive deficits?	–	+	++
Other elements of health care	…involve patient education and counseling specific for the cognitive deficits?	++	–	++
Information management	…connect patient-specific and evidence-based information concerning the cognitive deficits?	–	++	++

*+ denotes that this treatment approach takes the respective dimension of individualized treatment into account*.

*++ denotes that this treatment approach takes the respective dimension of individualized treatment into account very well*.

*– denotes that this treatment approach does not take the respective dimension of individualized treatment into account*.

## Central Cognitive Processes and Assessment of Executive (Dys)functions

In children with previous and ongoing severe acute or chronic illnesses, deficits in cognition can arise due to a number of factors. On the one hand, such diseases can directly affect the central nervous system and lead to structural or functional alterations which then impair cognitive functions and behavioral control. On the other hand, the structure and function of the central nervous system in these children can also be negatively affected by treatment, i.e., as side effects of pharmacological interventions, radio- and chemotherapy, and prolonged periods of hospitalization (20, 21). Depending on disease characteristics, developmental aspects (esp. neuronal plasticity) and treatment modalities, such impairments can be temporary or permanent. A large variety of cognitive functions, including attention, language, memory, and executive functions can be affected in children who have survived a serious illness. Based on their overarching significance for academic, vocational, and social functioning (22, 23), this paper will focus on executive function deficits occurring as a result of severe somatic illnesses in childhood (24).

Although a variety of (in some aspects even contrasting) models exist concerning executive functions, there is a general consensus that executive functions describe the ability to acquire, understand, and apply abstract and complex knowledge and to plan, direct, and control actions (24, 25). Poor executive functions have been associated with higher rates of violent crimes (26), risky financial behaviors (27), low social autonomy (28), and a generally lower quality of life (29). Executive functions can be examined using behavioral observations and questionnaire ratings [e.g., the behavior rating inventory of executive function or the learning, executive, and attention functioning scale (30)] and through specific neuropsychological tests like the behavioral assessment of the dysexecutive syndrome in children (31).

## Cognitive Training Approaches—Current Practice and Caveats

Once executive dysfunctions have been identified in the individual child, specific neurocognitive training programs can be used to ameliorate such deficits (22) from preschool age onwards. Diamond and Lee (32) review important influencing factors and crucial general characteristics of potential interventions in childhood in great detail. Such programs focus on (i) the restoration of existing impairments using specific training exercises, (ii) the acquisition of specific compensatory techniques related to the organization and planning of behavior, and (iii) the modification of environmental factors (23). The few empirical studies that have examined the effects of cognitive interventions in children with severe acute or chronic disorders show mixed results (33, 34). Despite some promising reports of improvements in trained domains such as attention and memory, inhibitory control as a central element of executive function was not improved after any of the examined interventions (33). In addition, and although such interventions often have a high ecological validity, the transfer to other, even closely related (untrained) cognitive/executive functions is limited (34). Also, the quality of the available studies is rather low (33). Most importantly, the pathophysiological and neurobiological pathways leading to the deficits or to any positive treatment outcomes are only taken into account to a limited extent (33) (see Figure 1). Such a more neurobiological, mechanistic viewpoint on the underlying processes may significantly aid the development of interventions that are targeted and individually adjustable [i.e., conform with approaches of individualized medicine (5)] (see Table 1).

## Dopamine-Based Mechanisms Underlying Executive Functions

Concerning neurobiological mechanisms, executive functions are mainly modulated by the catecholaminergic and dopaminergic system affecting fronto-striatal circuits (35, 36), even though serotonergic and glutamatergic mechanisms are also very important. Broadly speaking, “optimal” performance in numerous aspects of executive functions is reached when intermediate levels of dopamine are available (37, 38). In the case of “optimal” intermediate dopamine concentrations, there is an intricate balance between striatal dopamine D2-receptor related neural transmission (relevant for cognitive flexibility) and prefrontal dopamine D1-receptor related neural transmission (relevant for cognitive stability) (39). This pattern leads to an efficient balance between cognitive processes characterized by flexibility (e.g., readily allowing the processing of new sensory information driven by bottom-up influences) and stability (e.g., reducing distractibility and being driven by task goals in a top–down manner). Overall, cognitive flexibility and stability and the balance between them are crucial for executive functions (40).

## Pharmacological Interventions—Current Practice and Caveats

Consequently, pharmacological interventions modulating these dopaminergic abnormalities are frequently used in the treatment of several psychiatric symptoms or disorders. Selective dopamine D2 antagonists (neuroleptics) like Risperidone (41, 42), for example, reduce the flexibility of mental representations upon administration. On the other hand, methylphenidate (MPH), a dopamine reuptake inhibitor commonly used to treat children with attention deficit/hyperactivity disorder, prolongs the availability of dopamine in the synaptic cleft and leads to stronger activation of dopamine D1 receptors. This leads to more stable mental representations, and thus provides a balance for the D2-mediated cognitive flexibility. MPH has previously been applied within the context of disorders like cancer (43–45), sickle cell disease (46), and neurofibromatosis type 1 (47).

While these approaches are characterized by good ecological validity, are readily accessible and carry low costs for families and health care providers, they are not without problems (see Figure 1). For one, studies have not consistently shown significant and beneficial effects in these cases. MPH-related increases of general IQ levels were reported in children with neurofibromatosis type 1 (47). Moreover, positive effects of low doses (0.3 mg/kg) of MPH on attention and social functioning in survivors of brain tumors and leukemia in childhood (44) were found, but do hardly generalize to academic performance and success (45, 48). Similarly, children with sickle cell disease showed isolated MPH-related improvements in inhibitory control, while other executive functions and behavior resembling symptoms of attention-deficit hyperactivity disorder (ADHD) were not improved (46). Such mixed effects and the limited generalization may be explained by the fact that pharmacological interventions do not take the disease-specific cognitive profile and pathophysiological background into account, but function in a rather general fashion.

Thus, pharmacological interventions aiming to address cognitive (executive function) deficits in survivors of severe illnesses are connected to a trial-and-error approach and to *post hoc* hypotheses about why something has or has not been effective. This can lead to side effects concerning other cognitive functions and may interfere with pharmacological strategies addressing the somatic manifestations of the underlying disease. For example, pharmacological interventions targeting executive function deficits can induce cardio-metabolic changes and lowered arousal in the case of neuroleptics (49), and can lead to sleep problems, weight loss, and mood changes in the case of MPH (50). Therefore, medication may not always represent a practicable solution in clinical practice, also because it can, at least currently, hardly be tailored to the individual patients (see Table 1).

## Neuronal Oscillations and Their Relevance for Executive Functions

As outlined above, both neuropsychological training approaches and pharmacological interventions are unable to address the individually different pattern of executive dysfunctions and the underlying pathophysiological background to a sufficient extent (see Table 1). In this regard, the use of treatment strategies targeting neuronal oscillations represents a possible and highly attractive option. The term “neuronal oscillations” refers to the synchronized and rhythmic electrical brain activity that can be measured on the head using EEG electrodes (51). Although there still are significant gaps in scientific knowledge concerning their functional relevance, some neuronal oscillations have already been linked to some specific cognitive functions [e.g., See Ref. (52)]. The theta frequency band (4–7 Hz), for example, seems to play one of the most important roles, as it has been shown to be closely linked to the level of cognitive control (53–55). Specifically, theta oscillations originating from medial frontal regions (e.g., the anterior cingulate cortex) have been associated with attentional and cognitive resource allocation (53). Johnson et al. (56) have demonstrated that theta oscillations are an essential element of bidirectional connections between prefrontal and parietal regions and are highly relevant for working memory processes. Thus, the suggested connection to executive functions is at least partially mediated via long-range network connectivity, for which synchronized theta oscillations play a central role (57, 58). Conversely, executive functions are functionally largely based on precisely such long-range networks (54, 59, 60). In addition to frontal-midline theta power, beta oscillations (15–20 Hz) are also a relevant element as they have been suggested to be reflective of the maintenance of the current cognitive state and thus likely also represent elements of alertness and attention (61). Further, alpha oscillations should also be considered, since they have been suggested to represent the controlled access to information stored in memory (56, 62) and thus are also relevant for executive functions. Importantly, in all cases, a variety of oscillation characteristics apart from the frequency per se, such as power/amplitude, cross-frequency coupling, and phase locking, need to be considered in this context (51, 63, 64). Due to space limitations in the current paper, the interested reader is referred to a number of excellent reviews concerning neuronal oscillations in general and their relevance for executive functions in particular (53, 63, 65–68). We propose that—if interpreted carefully and handled critically—this available and constantly expanding knowledge could be beneficial when attempting to ameliorate executive function deficits in children and (young) adults with severe acute or chronic conditions.

## Oscillation-Based Interventions for Executive Dysfunctions: Neurofeedback

Such attempts have already been successfully made in the treatment of various pediatric (and adult) neuropsychiatric conditions in which neuronal oscillations have been shown to be altered [e.g., see Ref. (69, 70)]. One possibility of influencing these neuronal oscillations (and thus hopefully also the corresponding cognitive functions and behavior) is the application of frequency band neurofeedback training (71–73). Frequency band neurofeedback uses single EEG electrodes to record neuronal oscillations from the patient’s head. Via specifically designed software, these recordings are converted into animations or simple computer games (e.g., a car driving on a race course). The patient is able to control these games by regulating the relevant parameters of the EEG (i.e., the target oscillation). Neurofeedback is usually conducted over a period of 2–3 months with 1–2 weekly sessions. Originally, frequency band neurofeedback has been applied in the treatment of patients with ADHD, where a downregulation of theta activity and the complementary upregulation of beta activity is usually conducted (72, 74) and has shown encouraging effects on the level of symptoms (73, 75) and executive functions (71). Yet, it is likely that the efficacy of these protocols can be enhanced by training patients to upregulate theta power (76).

Importantly, depending on the individual profile of executive function problems, a similar approach may be applicable to patients, in whom the deficits stem from severe underlying somatic disorders or their treatment (see Figure 1). In fact, beneficial effects of frequency-based neurofeedback on self-reported cognitive measures have already been demonstrated in a sample of breast cancer survivors who suffered from cognitive impairments following treatment (77). Also, successful applications in patients with epilepsy (78) and chronic fatigue (79) have been reported. In contrast, one study has reported no significant differences between neurofeedback and placebo feedback training in pediatric brain tumor survivors (80). In this context, however, only beta oscillations or sensorimotor rhythms (12–15 Hz) were targeted, not taking theta frequencies, which play a crucial role in executive functioning, into account.

## Oscillation-Based Interventions for Executive Dysfunctions: Transcranial Stimulation Techniques

A less established approach may relate to the application of transcranial alternating current stimulation (tACS). Through tACS, a weak electrical current (<2 mA) is repeatedly applied to the head of the patient at the desired current frequency (e.g., 6 Hz). The assumption is that, after repetitive “training,” the brain will adapt to and “take over” these externally delivered oscillations (81). tACS theta frequency stimulation over parietal regions has been shown to result in significantly increased working memory capacity in healthy adults (82). So far, a small number of tACS-intervention studies have been conducted in patients with neurological disorders like epilepsy (83) and Parkinson’s disease (84), but studies reporting long-term effects or the applicability in pediatric populations have not been published so far. Overall, tACS has been described as a safe, specific, and direct possibility to influence pathological changes in neuronal oscillations (81). Yet, to be efficient, the targeted executive function deficit needs to be directly relatable to the characteristics (amplitude, frequency, phase, or regional coherence) of a particular neuronal oscillation (i.e., the stimulation protocol needs to be hypothesis-driven) and the tACS-induced changes must be present after stimulation offset (81). For these reasons and since the functional relevance of the oscillations in question remains to be investigated further, tACS should be classified as a rather experimental, but promising approach at this stage. Interestingly, another neuromodulation approach (transcranial direct current stimulation (tDCS)) has recently been used in conjunction with a short behavioral training, with this specific combination of interventions leading to (i) significant working memory improvements and (ii) changes in theta/alpha oscillations in a small group of healthy controls (85). Although its effects on neuronal oscillations may be of a more indirect or supportive nature, tDCS could thus also constitute a promising and safe (86) approach in this regard.

## Challenges, Arising Possibilities, and Future Perspectives for Oscillation-Based Treatment Approaches

Approaches like frequency band neurofeedback or tACS/tDCS could thus potentially be used to up-/downregulate patient’s oscillatory activity in order to achieve changes in cognition and thus also in behavior. However, there are also a number of challenges associated with this approach. For example, it may be argued that norm values for the power of the different frequency bands are only very difficult to obtain (74). This is the case, since such values vary considerably inter- and intraindividually and also strongly depend on the conditions under which they are measured. Moreover, basing intervention protocols for patients on oscillatory power measured in healthy individuals could lead to the use of training protocols which may not be as efficient as presumed (76), since the neurophysiological characteristics of a patient are probably different due to the mere presence of a disease. These individual differences need to be addressed using individually adapted intervention approaches. Decisions about which frequency bands should be up-/downregulated have to be based on the individual profile of executive (dys)function and on the (expanding) knowledge about the oscillatory correlates of these functions (see Figure 1).

Overall, we envisage that a promising and effective clinical approach to executive function deficits occurring in survivors of severe acute or chronic illnesses involves the development and application of specific, individualized treatment possibilities. Precisely, this could take place using oscillation-based intervention approaches. Currently, however, high initial costs (mainly for the equipment) and the resulting limited accessibility represent significant challenges for implementation. This is especially the case in more rural regions with a less developed medical infrastructure. Yet, a very effective way of addressing both of these challenges at once are “e-health” and “home-treatment approaches,” which would be particularly feasible in the case of neurofeedback training. To achieve this, EEG amplifiers are being developed that are portable, robust, and cost-effective due to only a few inbuilt electrode channels necessary for neurofeedback. In the future, such home-treatment approaches could be used to apply the feedback using the patients’ own mobile devices or TV/computer screens in their home environment. Such neurofeedback systems could be used to either supplement treatment sessions taking place in clinical settings or within the framework of telehealth initiatives, in which the entire course of treatment takes place in the home environment of the patient. Either way, to adhere to best practice and ethical standards (87) and to avoid or address side effects should they occur (88), such approaches would need to involve the close support and monitoring by clinicians experienced in the field. This is necessary, since such novel treatment approaches are only suitable for motivated patients in whom the current psychosocial situation is stable and who do not have any immediately threatening health concerns. To establish such boundary conditions, it may in some cases be helpful to initially attempt to target major cognitive deficits in attention or impulse control using pharmacological strategies described above. Such a procedure has been used successfully in patients with ADHD (71).

Furthermore, elements from cognitive-behavioral therapy (and in fact also the aforementioned cognitive training approaches) are an essential part of neuronal oscillation-based treatment approaches (72, 85). In addition, for home-treatment approaches, families need to be provided with a detailed introduction to the technical aspects of the treatment (e.g., how to set up the system, connect electrodes, conduct troubleshooting). To account for all these factors, at least one initial direct contact between the clinician and the patient family as well as regular follow-up contacts (potentially via phone or secure online platforms) are necessary. In addition, there also are ethical boundaries to consider in the case of home-treatment approaches that need to be monitored closely (e.g., financial constraints, involvement of health care provider and insurances, treatment offered by non-healthcare proficient sources). Finally, it remains to be clarified how any treatment outcomes could be effectively measured and quantified. This could potentially be achieved through the use of standardized cognitive test batteries which carry a high ecological validity.

So far, only one study has reported some outcomes of a partial neurofeedback home-treatment (89). Unfortunately, no control treatment was used and reported statistical analyses are limited to descriptions of individual symptom trajectories. This study, however, incorporates a detailed methodological description, and discusses a number of important considerations as well as possible pitfalls. More recently, alpha-frequency neurofeedback using a portable device has been shown to improve memory in a group of healthy adults (90). In addition, some home-treatment approaches have been reported concerning other biological parameters like electromyographic measures (91) and gait (92). Although these studies only report preliminary results, they nevertheless encourage further developments of these technologies. Through such developments, oscillations-based treatment options would not only become less costly, more widely available, and easy-to-use, but would also significantly enhance transferability of training success to daily life.

## Summary

In sum, we postulate that survivors of severe pediatric illnesses could greatly benefit from oscillation-based intervention approaches as they have the potential to ameliorate executive dysfunctions, thus leading to significant improvements in daily life. To benefit from the effects of neuronal plasticity, such approaches should be considered as early on in development as possible. Based on their high functional relevance for executive functions and the experience gained from interventions in areas like ADHD, targeting theta oscillations via neurofeedback could be a good starting point. Such approaches would provide new opportunities for the treating clinicians as they would close existing gaps between (pediatric) psychology, (neuro)pediatrics, and basic research, consequently leading to better clinical care for the affected children, (young) adults, and their families.

## Author Contributions

All authors conceived the paper, wrote and edited the manuscript and figure, and have approved the final version of this paper.

## Conflict of Interest Statement

AB, MH, and BN declare no competing or potential conflicts of interest. VR has received payment for consulting and writing activities from Lilly, Novartis, and Shire Pharmaceuticals, lecture honoraria from Lilly, Novartis, Shire Pharmaceuticals, and Medice Pharma, and support for research from Shire and Novartis. He has carried out (and is currently carrying out) clinical trials in cooperation with the Novartis, Shire, and Otsuka companies. CB has received payment for consulting from GlaxoSmithKline and Teva.
